# 

**Published:** 1886-03

**Authors:** 


CONTENTS

Vol. 33 No. 3

| | PAGE. |
|---|---|
| The Dietetic Movement | 65 |
| Ventilation of Rooms for Sick | 68 |
| Cocoaine as an Anasthetic | 72 |
| Trichinosis | 73 |
| Do Impressions on the Mother effect the Unborn Child | 75 |
| How do Indians Know? | 76 |
| Beef Tea | 77 |
| Diphtheria | 78 |
| Results of Pasteur's Inoculation | 79 |
| The Drunkard's Nose | 81 |
| Milk Diet for Gout | 82 |
| Bright's Disease of the Kidneys | 83 |
| The Ethics of Pain | 84 |
| A Cold-Footed Lady | 85 |
| Ozoniferous Essence | 85 |
| Our Sleeping Rooms | 86 |
| A New Method of Medicine | 87 |
| Cure for Asthma | 88 |
| Management of Children | 89 |
| Following Old Masters | 90 |
| Miscellaneous | 91 |
| Home Department | 93 |

ADVERTISEMENTS OF HALF A PAGE AND OVER.

| | |
|---|---|
| Fellows' Hypo-Phosphates | I |
| Lactopeptine | II |
| Castoria | II |
| Horsford's Acid Phosphates | III |
| Caulocorea | III |
| Medicated Suppositories | IV |
| Health Jolting Chair | V |
| Perforated Paper | 3d page of cover |
| Dr. Starkey | 4th page of cover |

